# Associations between sleep duration and cardiovascular diseases: A meta-review and meta-analysis of observational and Mendelian randomization studies

**DOI:** 10.3389/fcvm.2022.930000

**Published:** 2022-08-11

**Authors:** Shanshan Wang, Zhexi Li, Xiaoyu Wang, Sheng Guo, Yujing Sun, Guohua Li, Chenhao Zhao, Wenhui Yuan, Meng Li, Xiaolei Li, Sizhi Ai

**Affiliations:** Department of Cardiology, Life Science Center, Heart Center, The First Affiliated Hospital of Xinxiang Medical University, Weihui, China

**Keywords:** Mendelian randomization, observational study, meta-review, sleep duration, cardiovascular disease

## Abstract

The associations between sleep duration and cardiovascular diseases (CVDs) have been explored in many observational studies. However, the causality of sleep duration and many CVDs, such as coronary artery disease (CAD), heart failure (HF), and stroke, remains unclear. In this study, we conducted a systematic meta-review and meta-analysis of the results of observational and Mendelian randomization (MR) studies to examine how sleep duration impacts the risk of CVDs. We searched articles published in English and before 10 September 2021 in PubMed, Web of Science, and Embase. The articles were screened independently by two reviewers to minimize potential bias. We combined the meta-analyses of observational studies and 11 MR studies and summarized evidence of the effect of sleep duration on the risk of CAD, HF, stroke, and cardiovascular and all-cause mortality. Results showed that (a) evidence is accumulating that short sleep duration is a causal risk factor for CAD and HF; (b) abundant evidence from observational studies supports that long sleep duration is associated with the risk of CAD, stroke, and mortality, and long sleep duration has no causal associations with stroke and CAD in the MR studies; the causation of long sleep duration and other CVDs should be further studied; and (c) emerging evidence indicates that an increase in hours of sleep is associated with a decreased risk of CAD. Finally, we discussed the underlying pathophysiological mechanisms underlying short sleep duration and CVDs and suggested that increasing sleep duration benefits cardiovascular health.

## Introduction

Cardiovascular diseases (CVDs) are the leading causes of morbidity and mortality worldwide, accounting for approximately one-third of all deaths in 2019; 80% of these deaths occurred in low- and middle-income countries (1, 2). Although advances in the diagnosis and treatment of CVDs have been made, the incidence of CVDs is still increasing (3, 4). This situation highlights the importance of prevention. Many environmental and lifestyle factors, such as air pollution, nighttime light, noise exposure, and smoking, are associated with the risk of CVDs (5–9). In recent decades, sleep has been recognized as an important factor associated with many health outcomes (10–13). Numerous observational studies have shown U- or J-shaped associations between sleep duration and CVDs, suggesting that short and long periods of sleep duration are associated with a high risk of CVDs (14–17). However, a randomized controlled trial (RCT) for uncovering the association between sleep duration and CVDs is difficult to conduct, and thus the causality between sleep duration and many CVDs remains unclear.

In recent years, Mendelian randomization (MR) studies have used genetic variants as instrument variables to mimic the RCT design, that is, genetic variants are randomly assigned to individuals before birth; this design can provide causal inferences about many disease outcomes (18, 19). Several pioneer MR studies (20–23) have explored the causal association between sleep duration and CVDs. Some studies (20, 21) have shown that short sleep duration is associated with the risk of CVDs, but other studies (22, 23) have shown no causal association between sleep duration and CVDs. Therefore, a meta-analysis of the results of MR studies is needed to clarify the causal association between sleep duration and CVDs.

In this study, we conducted a systematic meta-review and meta-analysis of observational and MR studies to establish evidence of association and causality between sleep duration and several CVDs (i.e., coronary artery disease, heart failure, and stroke) or mortality. We discussed potential pathophysiological mechanisms underlying sleep duration and CVDs and provided suggestions for the prevention of CVDs through the reliable management of sleep.

## Methods

Our meta-analysis was performed according to the Preferred Reporting Items for Systematic Reviews and Meta-Analysis (PRISMA) guidelines (24). The detailed checklists of PRISMA are listed in Supplementary Table 1. The protocol of this meta-review was registered in the International Prospective Register of Systematic Reviews (PROSPERO; Registration number: CRD42021284908)^1^.

Our meta-review searched the meta-analyses of observational studies and MR studies about associations between sleep duration and CVDs. CVD is a general term for many cardiovascular and cerebrovascular diseases, such as coronary artery disease (CAD), heart failure (HF), and stroke (25). CAD is the most common type of heart disease and is also known as coronary heart disease (CHD) or ischemic heart disease, and mainly includes stable angina, unstable angina, myocardial infarction (MI), or sudden cardiac death (26). Acute myocardial infarction is a clinical myocardial injury (defined as an elevated cardiac troponin level; at least above the 99% upper reference limit) with myocardial necrosis (27). For HF, we mainly included chronic and stable HF and excluded acute decompensated HF. Stroke refers to ischemic and hemorrhagic strokes that are fatal or non-fatal, with no emphasis on initial or recurrent events. We included studies that investigated the association between sleep duration and all-cause and cardiovascular mortality. Three databases, namely, PubMed, Web of Science, and Embase, were searched for articles published in English and before 10 September 2021. The search words were as follows: (“sleep duration”) and (“cardiovascular disease” or “coronary artery disease” or “coronary heart disease” or “angina pectoris” or “myocardial infarction” or “heart failure” or “stroke” or “mortality”). In addition, we further reviewed the references of the articles to search for other relevant articles.

### Eligibility criteria of the included studies

The meta-analyses of observational studies (cross-sectional or prospective cohort studies) and MR studies that investigated correlation and causality between sleep duration and CVDs were included. If multiple meta-analyses investigated the same CVD, we used the latest published article. If a previous meta-analysis included more studies or contained novel findings that were not present in the latest article, this meta-analysis was also included. In the meta-analysis of MR studies, if two or more studies used the same database, we used the study with the largest sample size; if MR studies were not included in the meta-analysis, we described the results of these studies in this meta-review.

The exclusion criteria were as follows: (a) animal studies; (b) articles without available data; (c) studies on infants or children; and (d) articles that were not publicly published. The titles, abstracts, and full texts of the included articles were carefully examined by two independent reviewers (SW and ZL). If there were conflicts about the inclusion of articles between the two reviewers, a third reviewer (SA) evaluated unreconcilable disagreements.

### Data extraction

For the meta-analyses and MR studies, we extracted the odds ratio (OR), hazard ratio (HR), relative risk (RR), and 95% confidence interval (CI). If two or more MR studies investigated the same exposure factors (short or long sleep duration) and CVDs, we pooled the extracted ORs and 95% CIs. We also extracted the heterogeneity of each meta-analysis. The following data were obtained from each included article: author, year of publication, sample size (number of included studies), sleep duration categories (i.e., short and long sleep duration), CVD outcomes, and conclusions.

### Quality assessment of included studies

The Assessment of Multiple Systematic Reviews (AMSTAR) scale (28, 29) was used in assessing the quality of the included meta-analyses. This assessment tool has 11 criteria, including whether a preliminary design scheme is provided, whether literature retrieval is comprehensive, whether publication status meets the inclusion and exclusion criteria, whether the characteristics and scientific value of an included study are clear, whether the study methodology is appropriate, whether publication bias is detected, and whether the relevant conflict of interest is described. An item was scored as “yes” (1 point), “no” (0 point), or “not applicable” (0 point), and the highest total score was 11. AMSTAR scores are divided into high (9–11), medium (5–8), and low-quality (0–4) scores. The quality of an included study increases with the score.

No uniform standard for evaluating the quality of MR studies has been established. Given that MR rests on three main assumptions, namely, (1) genetic variants are related to risk factors (i.e., sleep duration); (2) genetic variants are not associated with any other confounders; and (3) genetic variants affect outcomes (CVD) only through exposure, we evaluated the quality of the methods of the included studies according to these assumptions (30, 31). If the first assumption was verified by providing F statistics (one-sample MR study) or selected SNP with significant correlation in GWAS (two-sample MR study), the rate was “good.” If the first assumption was verified with other means, the rate was “moderate.” If relevant verification was not performed or reported, the rate was “poor.” The second assumption can be verified by testing and reporting the association between genetic instruments and confounding. The last assumption, which is the exclusion–restriction assumption, states that genetic instruments cannot affect outcomes through factors other than interest exposure, also known as horizontal pleiotropy. For the second and third assumptions, SNP with pleiotropy should be excluded. Pleiotropy is mainly tested by MR-Egger regression and MR-PRESSO. We rated an article as “good” if both the second and third assumptions were tested, “moderate” if pleiotropy was only addressed on the basis of assumptions in other studies, or “poor” if pleiotropy was not evaluated.

### Statistical analysis

All statistical analyses were performed using R software (version 4.0.4; R Core Team, Vienna, Austria). The OR values of the MR studies that needed to be analyzed were pooled using a random-effects model, and the pooled results were evaluated using forest plots. Heterogeneity across studies was assessed using the *I*^2^ statistic and was considered mild when *I*^2^ was between 25% and 50%, moderate when *I*^2^ was between 50% and 75%, and severe when *I*^2^ was greater than 75%. A two-sided *P*-value of <0.05 was considered statistically significant.

## Results

### Literature search and study selection

**FIGURE 1:**
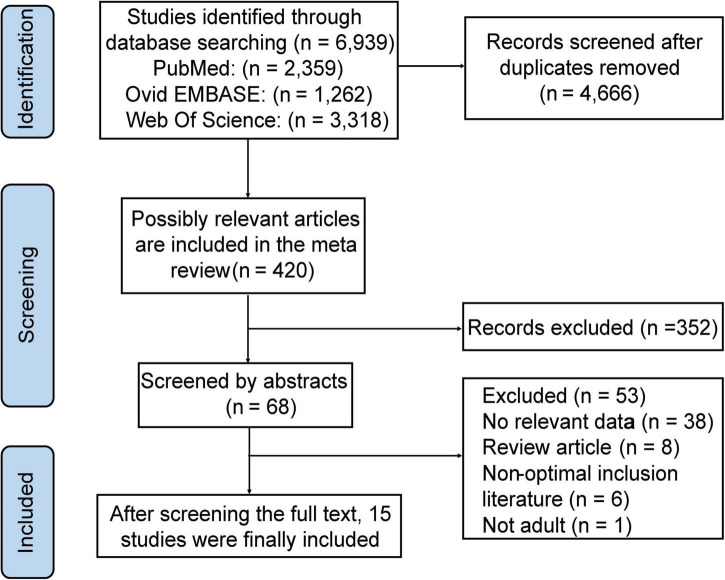
Flowchart of the records retrieved, screened, and included in the meta-review.

A total of 12,616 articles were searched in the three databases, of which 4,666 articles had no duplicates. After the initial screening of article titles and abstracts, 68 articles were considered relevant to this meta-review. Finally, 15 studies were included in the meta-review after full-text evaluation, of which four articles were meta-analyses and 11 articles were MR studies (Figure 1). As for CVD outcomes, six articles were related to CAD (20–23, 32, 33), three articles were related to HF (34–36), five articles were associated with stroke (20, 37–40), and two articles involved mortality (41, 42).

### Assessment of the quality of the included studies

The detailed scoring and final scores of each included meta-analysis are summarized in Supplementary Table 2. The AMSTAR scores of the four meta-analyses were 10 points (33), 8 points (38), 7 points (41), and 10 points (42). Supplementary Table 3 summarizes the validation of the MR assumptions of each included study. Regarding the validation of the MR hypothesis for each study, eight of the 11 MR studies (20, 21, 32, 35–37, 39, 40) included (70%) tested all three hypotheses, two tested hypothesis 1 only (23, 34), and one tested hypothesis 2 and 3 (22).

**FIGURE 2:**
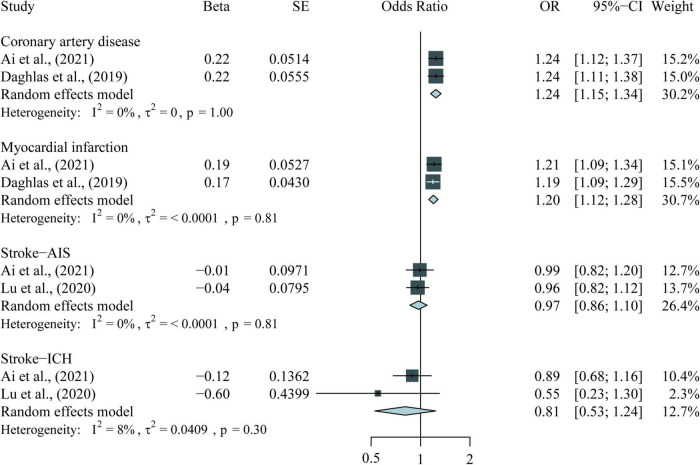
Meta-analysis results for the association between short sleep duration and CVDs in MR studies. CVDs, cardiovascular diseases; MR, Mendelian randomization; AIS, any ischemic stroke; ICH, primary intracranial hemorrhage.

**FIGURE 3:**
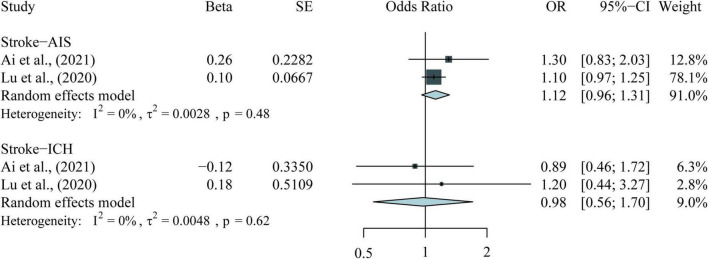
Meta-analysis results for the association between long sleep duration and CVDs in MR studies. CVDs, cardiovascular diseases; MR, Mendelian randomization; AIS, any ischemic stroke; ICH, primary intracranial hemorrhage.

**FIGURE 4:**
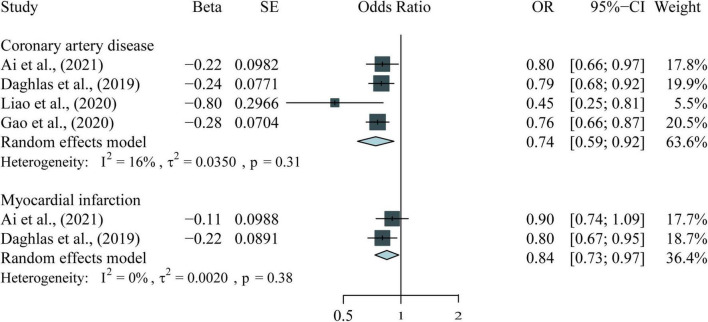
Meta-analysis results for the association between per hour longer sleep duration and CVDs in MR studies.

The following results combine four observational meta-analyses, 11 MR studies, and the meta-analysis of the included MR studies. Tables 1, 2 and Figures 2–4 provide the details of the meta-analysis of observational and MR studies.

**TABLE 1:** Sleep duration and cardiovascular diseases in observational meta-analyses.

References	Outcome	*n*	Exposure	Main results	Summary
Wang et al. (33) (AMSTAR = 11)	CHD	17	Short and long sleep duration	RR _reduction of 1 h per day_ = 1.11; 95% CI: 1.05–1.16 RR _increment of 1 h per day_ = 1.07; 95% CI: 1.00–1.15 compared with 7 h sleep duration per day	High-quality meta-analyses showed that both short and long sleep duration were significantly associated with increased risk of CHD, with high heterogeneity. Subgroup and sensitivity analysis further confirmed the above views.
He et al. (38) (AMSTAR = 9)	Stroke	16	Short and long sleep duration	Took 7 h of sleep as the reference: RRs (95% CI): 4 h: 1.17 (0.99–1.38); 5 h: 1.17 (1.00–1.37); 6 h: 1.10 (1.00–1.21); 8 h: 1.17 (1.07–1.28); 9 h: 1.45 (1.23–1.70); 10 h: 1.64 (1.4–1.92)	Equitable quality reviews showed that long sleep duration significantly increased the risk of overall stroke and fatal stroke in a linear manner.
Liu et al. (41) (AMSTAR = 8)	Mortality	40	Short and long sleep duration	Took 7 h of sleep as the reference: RRs (95% CI): 4 h: 1.05 (1.02–1.07); 5 h: 1.06 (1.03–1.09); 6 h: 1.04 (1.03–1.06); 8 h: 1.03 (1.02–1.05); 9 h: 1.13 (1.10–1.16); 10 h: 1.25 (1.22–1.29); 11 h: 1.38 (1.33–1.44)	Equitable quality reviews showed that both short and long sleep duration increased the risk of all-cause mortality, long sleep duration associated with a higher risk than short sleep duration, and the association was stronger in women. Sensitivity analysis further verified the reliability of the conclusion.
Pienaar et al. (42) (AMSTAR = 11)	Mortality	5	Short and long sleep duration	Short sleep duration: ACM: RR = 1.16; 95% CI: 1.11–1.22; *I*^2^ = 45.8% CVDM: RR = 1.26; 95% CI: 1.12–1.41; *I*^2^ = 45.8% Long sleep duration: ACM: RR = 1.18; 95% CI: 1.12–1.23; *I*^2^ = 86.0% CVDM: RR = 1.10; 95% CI: 0.95–1.27; *I*^2^ = 0.0%	High-quality meta-analyses showed that short sleep duration was significantly associated with both all-cause mortality and cardiovascular mortality, heterogeneity was not significant. The study focused on employed people under the age of 65 living in cities, highlighting the need for adequate sleep in the urban workforce.

AMSTAR, assessment of multiple systematic reviews; n, number of comparisons; CHD, coronary heart disease; RR, risk ratio; CI, confidence interval; ACM, all-cause mortality; CVDM, cardiovascular disease mortality.

**TABLE 2:** Causal relations of sleep duration and cardiovascular diseases in Mendelian randomization studies.

References	Outcomes	Sample	Exposure	Main results	Summary
(20)	CAD MI Stroke	Short sleep duration: *n* = 13,760 cases/66,110 controls and long sleep duration: *n* = 5629 cases/66,110 controls from Dashti et al.’s GWAS (43)	Short (≤6 h) and long (≥9 h) sleep durations	Short sleep duration: CAD: IVW OR = 1.24, 95% CI: 1.12–1.37; *P* = 4.09E-05 MI: IVW OR = 1.21, 95% CI: 1.09–1.34; *P* = 3.81E-04 AIS: IVW OR = 0.99, 95% CI: 0.82–1.20; *P* = 0.904 ICH: IVW OR = 0.89, 95% CI: 0.68–1.16; *P* = 0.399 Long sleep duration: CAD: IVW OR = 0.88, 95% CI: 0.69–1.34; *P* = 0.340 MI: IVW OR = 0.94, 95% CI: 0.73–1.22; *P* = 0.640 AIS: IVW OR = 1.30, 95% CI: 0.83–2.03; *P* = 0.245 ICH: IVW OR = 0.89, 95% CI: 0.46–1.71; *P* = 0.726 Per hour increase in sleep duration MI: IVW OR = 0.90; 95% CI: 0.74–1.09; *P* = 0.268 CAD: IVW OR = 0.80; 95% CI: 0.66– 0.97; *P* = 0.021	Linear and non-linear MR studies consistently show that genetically predicted short sleep duration has a causal and adverse effect on many CVDs, while genetically predicted long sleep duration has no association with the risk of most CVDs. Complementary analyses provided further evidence to support the results.
(21)	MI CAD	Short sleep duration: *n* = 106,192 cases/305,742 controls and long sleep duration: *n* = 34,184 cases/305,742 controls from Dashti et al.’s GWAS (43); MI: *n* = 43,676 cases/128,199 controls and CAD: *n* = 60,801 cases/123,504 controls from Nikpay et al.’s GWAS (84)	Short (<6 h) and long (> 9 h) sleep duration	Short sleep duration: MI: IVW OR = 1.19; 95% CI: 1.09–1.29; *P* = 4.2e-04 CAD: IVW OR = 1.24; 95% CI: 1.11–1.38; *P* = 1.79e-06 Per additional hour of sleep MI: IVW OR = 0.80; 95% CI: 0.67–0.95; *P* = 0.013 CAD: IVW OR = 0.79; 95% CI: 0.68–0.92; *P* = 3.20e-03	Two-sample MR studies found a dose-dependent causal association between short sleep duration and MI and CAD. Due to the limited NUMBER of SNPS associated with long sleep duration, no analysis was conducted. The results remained consistent after adjusting for confounding factors.
(32)	CHD	Short sleep duration: *n* = 106,192 cases/305,742 controls and long sleep duration: *n* = 34,184 cases/305,742 controls from Dashti et al.’s (43) GWAS; CHD: *n* = 60,801 cases/123,504 controls from Zhu et al.’s GWAS (85)	Sleep duration short (<7 h) and long (≥9 h) sleep duration	Sleep duration: OR = 0.755; 95% CI: 0.658–0.867 Short sleep duration: OR = 4.251; 95% CI: 2.396–7.541 Long sleep duration: OR = 0.208; 95% CI: 0.048–0.897	MR studies have confirmed that there is a causal association between sleep duration and CHD, short sleep duration increased the risk of CHD, but long sleep duration found no significant causal association.
(23)	CAD MI Stroke	Sleep durations: *n* = 47,180 from Gottlieb et al.’s GWAS (86); CAD: *n* = 60,801 cases/123,504 controls and MI: *n* = 43,676 cases/128,197 controls from Nikpay et al.’s GWAS (84); stroke: *n* = 37,792 cases/397,209 controls from SiGN’ s GWAS (87)	Sleep durations	CAD: IVW OR _per 1–SD higher in sleep duration_ = 1.00; 95% CI: 0.99–1.00; *P* = 0.162 MI: IVW OR _per 1–SD higher in sleep duration_ = 1.00; 95% CI: 1.00–1.00; *P* = 0.688 Stroke: IVW OR _per 1–SD higher in sleep duration_ = 1.00; 95% CI: 1.00–1.01; *P* = 0.231	There was no evidence of a causal association between sleep duration and CHD, MI, or stroke
(22)	CHD	Sleep durations: *n* = 335,410 from Neale Lab CHD: *n* = 15,420 cases/15,062 controls from Peden et al.’s GWAS [Coronary Artery Disease (C4D) Genetics Consortium, (88)]	Sleep durations	MR-Egger OR = 9.758; 95% CI: 0.160–592.894; *P* = 0.286 IVW OR = 0.451; 95% CI: 0.252–0.806; *P* = 0.007	The two-sample MR study did not find a causal association between sleep duration and CHD.
(36)	HF	Short sleep duration: *n* = 106,192 cases/305,742 controls and long sleep duration: *n* = 34,184 cases/305,742 controls from Dashti et al.’s GWAS (43). HF: *n* = 47,309 cases/930,014 controls from Shah et al.’s GWAS (44).	Short (<7 h) and long (≥9 h) sleep duration	Short sleep duration: IVW OR = 1.136; 95%; CI = 1.025–1.258; *P* = 0.015 Long sleep duration: IVW OR = 0.921; 95% CI = 0.813–1.045; *P* = 0.202	Two-sample MR study showed that genetically predicted short sleep duration increased the risk of HF, but there is no evidence of a causal association between long sleep duration and HF.
(35)	HF	Sleep durations: *n* = 446,118 from Dashti et al.’s GWAS (43). HF: *n* = 47,309 cases/930,014 controls from Shah et al.’s GWAS (44).	Short (<7 h) and long (≥9 h) sleep duration	Short sleep duration: IVW OR = 1.14; 99% CI: 0.97–1.33; *P* = 0.041 Long sleep duration: IVW OR = 0.85; 99% CI: 0.65–1.13; *P* = 0.149	MR study found that short sleep duration is a causal risk factor of HF, and longer sleep duration may reduce the risk of HF.
(43)	HF	*N* = 30,251 from Karlson et al.’s GWAS (89).	Short (<7 h) and long (≥9 h) sleep duration	Longer sleep duration with congestive heart failure: IVW OR _per minute of sleep_ = 0.978; 95% CI: 0.961–0.996; *P* = 0.019	Evidence suggested a causal association between genetically predicted longer sleep duration and reduced risk of HF, sensitivity analyses have a consistent effect.
(40)	Stroke	Short sleep duration: *n* = 106, 192 cases/305,742 controls and long sleep duration: *n* = 34,184 cases/305,742 controls from Dashti et al.’s GWAS (43); stroke: *n* = 40,585 cases/406,111 controls from Malik et al.’s GWAS (90) and *n* = 1,681 cases/2,261 controls from Daniel Woo et al.’s GWAS (91).	Short (<7 h) and long (≥9 h) sleep duration	Short sleep duration: All stroke: IVW OR = 1.13; 95% CI: 1.00–1.27; *P* = 0.052 AIS: IVW OR = 1.10; 95% CI: 0.97–1.26; *P* = 0.142 LAS: IVW OR = 1.41; 95% CI: 1.02–1.95; *P* = 0.040 CES: IVW OR = 0.99; 95% CI: 0.74–1.32; *P* = 0.938 SVS: IVW OR = 1.02; 95% CI: 0.75–1.37; *P* = 0.911 Long sleep duration: All stroke: IVW OR = 0.91; 95% CI: 0.78–1.07; *P* = 0.252 AIS: IVW OR = 0.97; 95% CI: 0.82–1.15; *P* = 0.723 LAS: IVW OR = 1.25; 95% CI: 0.82–1.91; *P* = 0.291 CES: IVW OR = 1.35; 95% CI: 0.97–1.88; *P* = 0.073 SVS: IVW OR = 1.05; 95% CI: 0.71–1.56; *P* = 0.786	This study found no causal association between short or long sleep duration and total stroke. Short sleep duration was associated with an increased risk of total ischemic stroke, but sensitivity analyses were less accurate.
(39)	Stroke	Short sleep duration: *n* = 106,192 and long sleep duration: *n* = 34,184 from Dashti et al.’s GWAS (43); stroke: *n* = 40,585 cases/406,111 controls from Malik et al.’s GWAS (90)	Short (<7 h) and long (≥9 h) sleep duration	Short sleep duration: All stroke: IVW OR = 0.91; 95% CI: 0.78–1.07; *P* = 0.25 AIS: IVW OR = 0.96; 95% CI: 0.82–1.12; *P* = 0.58 LAS: IVW OR = 1.26; 95% CI: 0.85–1.86; *P* = 0.26 CES: IVW OR = 1.33; 95% CI: 0.98–1.81; *P* = 0.07 SVS: IVW OR = 0.86; 95% CI: 0.56–1.34; *P* = 0.51 ICH: IVW OR = 0.55; 95% CI: 0.23–1.29; *P* = 0.17 Long sleep duration: All stroke: IVW OR = 1.13; 95% CI: 1.00–1.27; *P* = 0.05 AIS: IVW OR = 1.10; 95% CI: 0.97–1.26; *P* = 0.14 LAS: IVW OR = 1.41; 95% CI: 1.02–1.95; *P* = 0.04 CES: IVW OR = 0.99; 95% CI: 0.74–1.32; *P* = 0.94 SVS: IVW OR = 1.02; 95% CI: 0.75–1.37; *P* = 0.91 ICH: IVW OR = 1.20; 95% CI: 0.44–3.26; *P* = 0.72	Two-sample MR study found no causal association between long and short sleep duration with stroke and related subtypes.
(37)	Ischemic stroke and its subtypes	Short sleep duration: *n* = 106, 192 cases/305,742 controls and long sleep duration: *n* = 34,184 cases/305,742 controls from Dashti et al.’s GWAS (43); stroke: *n* = 34,217 cases/406,111 controls from Malik et al.’s GWAS (90)	Short (≤6 h) and long (≥9 h) sleep durations	Per doubling of genetic liability for short sleep duration: LAS: IVW OR = 1.27; 95% CI: 1.01–1.58; *P* = 0.038 SVS: IVW OR = 1.01; 95% CI: 0.82–1.25 CES: IVW OR = 0.99; 95% CI: 0.83–1.18 AIS: IVW OR = 1.07; 95% CI: 0.98–1.17 Long sleep duration: LAS: IVW OR = 1.17; 95% CI: 0.87–1.56; *P* = 0.291 SVS: IVW OR = 1.04; 95% CI: 0.79–1.36; *P* = 0.789 CES: IVW OR = 1.23; 95% CI: 0.98–1.55; *P* = 0.073 AIS: IVW OR = 0.98; 95% CI: 0.87–1.10; *P* = 0.723	Two-sample MR study provided suggestive evidence for a potential causal effect of short sleep duration on the risk of LAS, but not SVS, CES, or AIS. These results were overall robust to sensitivity analyses.

CAD, coronary artery disease; CHD, coronary heart disease; MI, myocardial infarction; MR, Mendelian randomization; GWAS, genome-wide association study; IVW, inverse-variance weighted; OR, odds ratio; CI, confidence interval; GRS, genetic risk score; SNP, single-nucleotide polymorphism; HF, heart failure; AIS, any ischemic stroke; LAS, large artery stroke; SVS, small vessel stroke; CES, cardioembolic stroke; ICH, primary intracranial hemorrhage.

### Association between sleep duration and cardiovascular diseases from observational meta-analyses

A prospective systematic review and meta-analysis (33) showed a U-shaped association between sleep duration and risk of CHD, indicating short and long periods of sleep were associated with an increased risk of CHD. The combined RR (95% CI) of CHD for a decrease and an increase of 1 h/day in sleep duration was calculated using a parameterized method. Compared with 7 h sleep per day, the RR of CHD was 1.11 (95% CI = 1.05–1.16, *I*^2^ = 58.9%) for each 1 h reduction and 1.07 (95% CI = 1.00–1.15, *I*^2^ = 70.5%) for each 1 h increase, but the heterogeneity was high. Subgroup analysis and sensitivity analysis showed that the study location and number of cases in each study may be the sources of heterogeneity (Table 1).

The association between sleep duration and stroke was examined in a meta-analysis with 16 cohorts and 528,653 participants (38). Non-linear analysis showed a J-shaped association between sleep duration and total stroke, and the lowest risk was found at 7 h sleep duration. When using 7 h sleep duration as a reference, every additional hour of sleep duration was associated with a 13% increase in total stroke (RR = 1.13, 95% CI = 1.07–1.20) and 12% increase in fatal stroke (RR = 1.12, 95% CI = 1.04–1.21) (Table 1).

Two meta-analyses (41, 42) examined the prospective association of sleep duration with all-cause and cardiovascular mortalities. The largest meta-analysis (41) included 40 cohorts with 2,200,425 participants and showed that a 1 h increase (RR = 1.12, 95% CI = 1.09–1.15) and decrease (RR = 1.07, 95% CI = 1.03–1.11) in sleep duration were associated with an increased risk of all-cause mortality when referenced to the mortality of 7 h sleep duration. Subgroup analysis found that the association was stronger in women. A recent meta-analysis and systematic review (42) reported similar results, showing that short sleep duration was associated with all-cause mortality (RR = 1.16, 95% CI = 1.11–1.22) and cardiovascular mortality (RR = 1.26, 95% CI = 1.12–1.41) compared with normal sleep duration (6–8 h/day) without significant heterogeneity, whereas long sleep duration was associated with all-cause mortality (RR = 1.18, 95% CI = 1.12–1.23) but not cardiovascular mortality (RR = 1.10, 95% CI = 0.95–1.27), the heterogeneity was high (*I*^2^ = 86.0; Table 1).

### Association between sleep duration and cardiovascular diseases from the meta-analysis of Mendelian randomization studies

A total of 11 MR studies were included in this study (Table 2). The results of the meta-analysis of short sleep duration and CVDs in the MR studies are shown in Figure 2. Genetically predicted short sleep duration was associated with an increased risk of CAD (OR = 1.24, 95% CI = 1.15–1.34, *P* < 0.001) and MI (OR = 1.20, 95% CI = 1.12–1.28, *P* < 0.001). The causal association between short sleep duration and HF was examined in two MR studies (34, 35), which used the same GWAS dataset (43, 44). Both studies suggested that genetically predicted short sleep duration is associated with the risk of HF. Four MR studies examined the causality between short sleep duration and stroke or its subtype (20, 37, 39, 40). Three of the studies (37, 39, 40) had GWAS data from the same source and showed no causal association between short sleep duration and all strokes, cardioembolic stroke (CES), or small vessel stroke (SVS). Moreover, no causation was found between short sleep duration and any ischemic stroke (AIS; OR = 0.97, 95% CI = 0.86–1.10, *P* = 0.64) and primary intracranial hemorrhage (ICH; OR = 0.81, 95% CI = 0.53–1.24, *P* = 0.33) in the meta-analysis with our previous study (20). Results for the causal association between short sleep duration and large artery stroke (LAS) (37, 39, 40) were inconsistent despite that these studies used the same GWAS dataset.

As for the long sleep duration, neither of the two MR studies (20, 32) found a causal association between long sleep duration and CAD. Regarding HF, two MR studies (35, 36) used the same GWAS data and found that long sleep duration had no association with the risk of HF. As for stroke, three studies (37, 39, 40) used the same GWAS data but showed inconsistent results. Lu et al. (39) found weak evidence of the causal association between long sleep duration and LAS (OR = 1.41; 95% CI: 1.02–1.95; *P* = 0.04), but two studies (37, 40) did not support this association. All three MR studies (37, 39, 40) did not find any causal association between long sleep duration and the risk of all stroke, CES, or SVS. Moreover, no causal association for AIS (OR = 1.12; 95% CI: 0.96–1.31; *P* = 0.15) and CIH (OR = 0.98; 95% CI: 0.56–1.70; *P* = 0.93) was found through meta-analysis in our previous study (Figure 3).

We also meta-analyzed the results of continuous sleep duration and CVDs in several MR studies (20–22, 32). There are significant negative associations of continuous sleep duration with CAD (OR = 0.74, 95% CI = 0.59–0.92, *P* < 0.001) and MI (OR = 0.84, 95% CI = 0.73–0.97, *P* = 0.02), indicating that per 1 h increase in sleep duration is associated with a lower risk of CVDs (Figure 4).

## Discussion

In this meta-review, evidence obtained by the meta-analyses of observational studies supported that short and long sleep duration were associated with the risk of CAD, stroke, and mortality. The MR studies and the related meta-analyses supported that short sleep duration rather than long sleep duration is associated with the risk of CAD or HF. The causal associations of short and long sleep durations with the risks of stroke and mortality need further investigation.

Previous observational studies have found that short sleep duration is associated with the risk of CVDs, such as CAD, HF, and stroke (45–47). Moreover, short sleep duration was associated with several cardiovascular risk factors, such as type 2 diabetes and overweight (48–50). Evidence obtained by the meta-analyses of the observational studies further confirmed that short sleep duration may be an independent risk factor for CHD, all-cause and cardiovascular mortalities (51–54). As for stroke, observational studies suggested that short sleep duration is associated with the risk of stroke (46, 55), and a meta-analysis of observational studies (56) further confirmed that short sleep duration was significantly associated with the risk of stroke. However, the most recent dose-response meta-analysis (38) showed that long rather than short sleep duration is associated with the risk of stroke. Different sample sizes and specific population composition in these studies may have contributed to the inconsistent results.

Mendelian randomization studies (21, 32) supported the potential causal association between short sleep duration and CHD. However, Zhuang et al. (23) and Liao et al. (22) reported inconsistent results for the causal association between short sleep duration and CHD. The limited number of genetic variants used in these studies may have reduced the statistical power of the MR estimates. Our MR study (20) further confirmed that short sleep duration is a potential causal risk factor for many CVDs. The underlying pathophysiological mechanisms, including sympathetic activation (57, 58), cardiac endocrine and metabolic dysfunction (11, 59), increased inflammatory mediators (60), and endothelial dysfunction (61, 62), may be involved in the adverse effects of short sleep duration on cardiovascular health. Recent studies have shown that extended sleep duration can reduce the risk of obesity and improve cardiovascular health, especially in people who have long-term sleep deprivation (49, 63–65). A recent meta-analysis provided preliminary evidence supporting that prolonging sleep duration can improve cardiometabolic health (66). Therefore, for short sleepers, extending sleep duration may be a potentially promising strategy for reducing the risk of CVDs.

Compared with short sleep duration, long sleep duration has more pronounced associations with many CVDs (15, 67). Observational studies have shown that long sleep duration is associated with the risk of CHD and all-cause and cardiovascular mortalities (68–70). The association between long sleep duration and stroke has been widely investigated in many observational studies (38, 54, 71–73). Moreover, a gender difference was found in this association; women who sleep longer have a higher risk of stroke (74). Another study (75) showed that long sleep duration is associated with the risk of hemorrhagic stroke but not ischemic stroke in women. However, MR studies (20, 36, 40) did not provide sufficient evidence supporting the causal association between long sleep duration and the risk of CVDs (i.e., CHD, HF, and stroke). No clear experimental evidence shows the harmful effects of long sleep duration on the cardiovascular system. Long sleep duration has been suggested to be associated with sleep apnea, unemployment, or low socioeconomic status (59, 76, 77). In addition, some other confounding factors, such as depression and neuroticism, may also impact the association between sleep duration and CVDs (78–81). Therefore, the observed significant associations between long sleep duration and CVDs in many observational studies may have been confounded by these unmeasured factors and reflected potential reverse causality. Long sleep duration may be a surrogate risk indicator for poor health status or sleep quality, which may also increase CHD risk (67, 82, 83). For people who have a long sleep duration, we should not directly recommend increasing sleep duration to reduce the risk of CVDs.

## Limitations

Several limitations in this meta-review should be considered. First, we accepted the conclusions of the meta-analyses of observational studies rather than performing a meta-analysis, and the limitations of the primary study might have been ignored. Second, the measurement of sleep duration in the original study mainly depended on questionnaires, and thus bias of measurement error may be present. Third, high heterogeneity was observed in some studies. Although most studies analyzed the possible sources of heterogeneity, the findings should be interpreted carefully. Fourth, we summarized the qualitative data of some studies to draw the association between sleep duration and CVDs. In this process, we may have misunderstood the proposal of an author or added too much of our own understanding of the data. Finally, the included studies were relatively few in some disease outcomes and mainly published in English. Recently published studies or studies published in other languages were not included in this meta-review.

## Conclusion

In summary, our meta-review found that short sleep duration may be a causal risk factor for many CVDs. Although observational meta-analysis supported that long sleep duration is associated with the risk of CVDs, the causal association between long sleep duration and CVDs remains unclear. Our results suggest that extending sleep duration in sleep-deprived people may be beneficial for their cardiovascular health.

## Data availability statement

The original contributions presented in this study are included in the article/Supplementary material, further inquiries can be directed to the corresponding author.

## Author contributions

SA and SW designed the protocol and drafted the manuscript. SW and ZL searched the databases and screened the retrieved manuscript. SG, XW, and YS extracted data and assessed study quality. GL and CZ analyzed the data. WY and XL prepared the figures. ML prepared the tables. All authors participated in the interpretation of the data and read and approved the final version to be published.

## Footnotes

1
https://www.crd.york.ac.uk/prospero/
