# Supplementary material for: Associations between sleep duration and cardiovascular diseases: A meta-review and meta-analysis of observational and Mendelian randomization studies
Source: Front Cardiovasc Med. 2022 Aug 11;9:930000. doi: 10.3389/fcvm.2022.930000 (PMC9403140; doi:10.3389/fcvm.2022.930000)
Supplement: Supplementary file 1 [file Table_1.DOCX]

Supplementary Information

**Associations between Sleep Duration and Cardiovascular Diseases: A Meta-review and Meta-analysis of Observational and Mendelian Randomization Studies**

| **Item No** | **Title** |
| --- | --- |
| Table 1 | PRISMA 2020 Checklist |
| Table 2 | AMSTAR scoring results of the included systematic reviews and meta- analysis |
| Table 3 | Validation of the 3 Assumptions of Mendelian Randomization in Each Study |

**Table S1.** PRISMA 2020 Checklist

| **Section and Topic** | **Item #** | **Checklist item** | **Location where item is reported** |
| --- | --- | --- | --- |
| **TITLE** | | |  |
| Title | 1 | Identify the report as a systematic review. | 1 |
| **ABSTRACT** | | |  |
| Abstract | 2 | See the PRISMA 2020 for Abstracts checklist. | 2 |
| **INTRODUCTION** | | |  |
| Rationale | 3 | Describe the rationale for the review in the context of existing knowledge. | 2 |
| Objectives | 4 | Provide an explicit statement of the objective(s) or question(s) the review addresses. | 2 |
| **METHODS** | | |  |
| Eligibility criteria | 5 | Specify the inclusion and exclusion criteria for the review and how studies were grouped for the syntheses. | 3 |
| Information sources | 6 | Specify all databases, registers, websites, organisations, reference lists and other sources searched or consulted to identify studies. Specify the date when each source was last searched or consulted. | 3 |
| Search strategy | 7 | Present the full search strategies for all databases, registers and websites, including any filters and limits used. | 3 |
| Selection process | 8 | Specify the methods used to decide whether a study met the inclusion criteria of the review, including how many reviewers screened each record and each report retrieved, whether they worked independently, and if applicable, details of automation tools used in the process. | 3 |
| Data collection process | 9 | Specify the methods used to collect data from reports, including how many reviewers collected data from each report, whether they worked independently, any processes for obtaining or confirming data from study investigators, and if applicable, details of automation tools used in the process. | 3 |
| Data items | 10a | List and define all outcomes for which data were sought. Specify whether all results that were compatible with each outcome domain in each study were sought (e.g. for all measures, time points, analyses), and if not, the methods used to decide which results to collect. | 3 |
|  | 10b | List and define all other variables for which data were sought (e.g. participant and intervention characteristics, funding sources). Describe any assumptions made about any missing or unclear information. | NA |
| Study risk of bias assessment | 11 | Specify the methods used to assess risk of bias in the included studies, including details of the tool(s) used, how many reviewers assessed each study and whether they worked independently, and if applicable, details of automation tools used in the process. | 3 |
| Effect measures | 12 | Specify for each outcome the effect measure(s) (e.g. risk ratio, mean difference) used in the synthesis or presentation of results. | 3 |
| Synthesis methods | 13a | Describe the processes used to decide which studies were eligible for each synthesis (e.g. tabulating the study intervention characteristics and comparing against the planned groups for each synthesis (item #5)). | Table 2 |
|  | 13b | Describe any methods required to prepare the data for presentation or synthesis, such as handling of missing summary statistics, or data conversions. | NA |
|  | 13c | Describe any methods used to tabulate or visually display results of individual studies and syntheses. | 4 |
|  | 13d | Describe any methods used to synthesize results and provide a rationale for the choice(s). If meta-analysis was performed, describe the model(s), method(s) to identify the presence and extent of statistical heterogeneity, and software package(s) used. | 4 |
|  | 13e | Describe any methods used to explore possible causes of heterogeneity among study results (e.g. subgroup analysis, meta-regression). | NA |
|  | 13f | Describe any sensitivity analyses conducted to assess robustness of the synthesized results. | NA |
| Reporting bias assessment | 14 | Describe any methods used to assess risk of bias due to missing results in a synthesis (arising from reporting biases). | NA |
| Certainty assessment | 15 | Describe any methods used to assess certainty (or confidence) in the body of evidence for an outcome. | 4 |
| **RESULTS** | | |  |
| Study selection | 16a | Describe the results of the search and selection process, from the number of records identified in the search to the number of studies included in the review, ideally using a flow diagram. | 4 and Figure 1 |
|  | 16b | Cite studies that might appear to meet the inclusion criteria, but which were excluded, and explain why they were excluded. | NA |
| Study characteristics | 17 | Cite each included study and present its characteristics. | Table 1 and Table 2 |
| Risk of bias in studies | 18 | Present assessments of risk of bias for each included study. | NA |
| Results of individual studies | 19 | For all outcomes, present, for each study: (a) summary statistics for each group (where appropriate) and (b) an effect estimate and its precision (e.g. confidence/credible interval), ideally using structured tables or plots. | Table 1 and Table 2 |
| Results of syntheses | 20a | For each synthesis, briefly summarise the characteristics and risk of bias among contributing studies. | NA |
|  | 20b | Present results of all statistical syntheses conducted. If meta-analysis was done, present for each the summary estimate and its precision (e.g. confidence/credible interval) and measures of statistical heterogeneity. If comparing groups, describe the direction of the effect. | Figure 1、2 and 3 |
|  | 20c | Present results of all investigations of possible causes of heterogeneity among study results. | NA |
|  | 20d | Present results of all sensitivity analyses conducted to assess the robustness of the synthesized results. | NA |
| Reporting biases | 21 | Present assessments of risk of bias due to missing results (arising from reporting biases) for each synthesis assessed. | NA |
| Certainty of evidence | 22 | Present assessments of certainty (or confidence) in the body of evidence for each outcome assessed. | NA |
| **DISCUSSION** | | |  |
| Discussion | 23a | Provide a general interpretation of the results in the context of other evidence. | 6 and 7 |
|  | 23b | Discuss any limitations of the evidence included in the review. | 7 |
|  | 23c | Discuss any limitations of the review processes used. | 7 |
|  | 23d | Discuss implications of the results for practice, policy, and future research. | 7 |
| **OTHER INFORMATION** | | |  |
| Registration and protocol | 24a | Provide registration information for the review, including register name and registration number, or state that the review was not registered. | 3 |
|  | 24b | Indicate where the review protocol can be accessed, or state that a protocol was not prepared. | 3 |
|  | 24c | Describe and explain any amendments to information provided at registration or in the protocol. | NA |
| Support | 25 | Describe sources of financial or non-financial support for the review, and the role of the funders or sponsors in the review. | 7 |
| Competing interests | 26 | Declare any competing interests of review authors. | 8 |
| Availability of data, code and other materials | 27 | Report which of the following are publicly available and where they can be found: template data collection forms; data extracted from included studies; data used for all analyses; analytic code; any other materials used in the review. | NA |

From: Page MJ, McKenzie JE, Bossuyt PM, Boutron I, Hoffmann TC, Mulrow CD, et al. The PRISMA 2020 statement: an updated guideline for reporting systematic reviews. BMJ 2021;372:n71. doi: 10.1136/bmj.n71

Table 2: AMSTAR scoring results of the included systematic reviews and meta- analysis

| References | Item 1 | Item 2 | Item 3 | Item 4 | Item 5 | Item 6 | Item 7 | Item 8 | Item 9 | Item 10 | Item 11 | Total score |
| --- | --- | --- | --- | --- | --- | --- | --- | --- | --- | --- | --- | --- |
| Wang et al., (2016) | Y | Y | Y | Y | Y | Y | Y | Y | Y | Y | Y | 11 |
| He et al., (2017) | Y | Y | Y | Y | NA | Y | Y | Y | Y | Y | N | 9 |
| Liu et al., (2017) | Y | Y | Y | Y | NA | Y | Y | Y | N | N | Y | 8 |
| Pienaar et al., (2021) | Y | Y | Y | Y | Y | Y | Y | Y | Y | Y | Y | 11 |

AMSTAR: Assessment of Multiple Systematic Reviews; Y: yes; N: No; NA: Not applicable; C: Can't answer. Item 1. Was an ‘a priori’ design provided? Item 2. Was there a duplicate study selection and data extraction? Item 3. Was a comprehensive literature search performed? Item 4. Was the status of publication (i.e., grey literature) used as an inclusion criterion? Item 5. Was a list of studies (included and excluded) provided? Item 6. Were the characteristics of the included studies provided? Item 7. Was the scientific quality of the included studies assessed and documented? Item 8. Was the scientific quality of the included studies used appropriately in formulating conclusions? Item 9. Were the methods used to combine the findings of studies appropriate? Item 10. Was the likelihood of publication bias assessed? Item 11. Was the conflict of interest stated?

Table 3: Validation of the 3 Assumptions of Mendelian Randomization in Each Study

| References | Assumption 1^a^ | Assumptions 2 and 3^b^ | Conclusion |
| --- | --- | --- | --- |
| Ai et al., (2021) | Association between gene and sleep duration has been estimated in previous studies (p<5×10^−8^; 43 loci at p<6×10^−9^). | MR-Egger were account for potential pleiotropy, and no pleiotropy was found. | All 3 assumptions validated; and suggested no directional pleiotropy for the CVD outcomes |
| Daghlas et al., (2019) | Same as Ref of Ai et al. | The MR Egger intercept test for horizontal pleiotropy was not significant (P _short sleep_ = 0.22). | All 3 assumptions validated; horizontal pleiotropy does not exist. |
| Gao et al., (2020) | Same as Ref of Ai et al. | HEIDI-outlier test and HEIDI-global test were used to test the pleiotropy, and no pleiotropy was found by both methods. | All 3 assumptions validated; and there is no pleiotropy. |
| Zhuang et al., (2020) | In discovery cohorts in two independent loci, gene significant association with sleep duration | Pleiotropy was not estimated. | Assumption 1 was validated. Pleiotropy was not tested for, so it could exist. |
| Liao et al., (2020) | Association between gene and sleep duration not tested for, only the database of the source is given. | Test indicated that there was no heterogeneity (P = 0.555) and horizontal pleiotropy (P = 0.150). | Assumption 1 not validated. No heterogeneity and horizontal pleiotropy |
| Zhao et al., (2021) | Same as Ref of Ai et al. | Potential pleiotropy of the genetic variants was accounted by MR-Egger method and found no horizontal pleiotropy (P _short sleep_ = 0.329; P _long sleep_ = 0.572). | All 3 assumptions validated; There showed no evidence of directional pleiotropy in the analyses |
| Doherty et al., (2018) | The relationship between genes and sleep duration is significant, which has been confirmed in previous studies. | MR-PRESSO method was used to evaluate horizontal pleiotropy. No pleiotropy in long sleep duration (P = 0.230), short sleep duration indicated pleiotropy (P = 0.041). | All 3 assumptions validated; Possible risk of bias due to pleiotropy in short sleep duration. |
| Dashti et al., (2019) | Same as Ref of Ai et al. | Pleiotropy was not detected or adjusted. | Only assumption 1 was validated. Due to the lack of detection for pleiotropy, deviation may exist. |
| Titova et al., (2020) | Same as Ref of Ai et al. | The MR-Egger method was used to assess directional pleiotropy. There was no evidence of directional pleiotropy. | All 3 assumptions validated; Directional pleiotropy was not found. |
| Lu et al., (2020) | Same as Ref of Ai et al. | MR-Egger regression and MR-PRESSO global tests demonstrated no horizontal pleiotropy. | All of the 3 assumptions were validated; Horizontal pleiotropy was identified by two methods. |
| Cai et al., (2020) | Same as Ref of Ai et al. | MR-Egger intercept tests suggested no horizontal pleiotropy (all P for intercept ≥ 0.445). | All 3 assumptions validated; There was no evidence of directional pleiotropy. |

^a^ Genotype must be associated with phenotype (sleep durations); validated in 9 studies.

^b^ Absence of pleiotropy (ie, genotype should not be associated with confounders and should affect outcome only through the risk factor); verified in 9 studies.

MR: Mendelian randomization; CVDs: Cardiovascular diseases

**REFERENCES**

Ai, S., Zhang, J., Zhao, G., Wang, N., Li, G., So, H.C., et al. (2021). Causal associations of short and long sleep durations with 12 cardiovascular diseases: Linear and nonlinear mendelian randomization analyses in uk biobank. *Eur Heart J.* 42, 3349-3357. doi: 10.1093/eurheartj/ehab170

Cai, H., Liang, J., Liu, Z., Fang, L., Zheng, J., Xu, J., et al. (2020). Causal effects of sleep traits on ischemic stroke and its subtypes: A mendelian randomization study. *Nat Sci Sleep.* 12, 783-790. doi: 10.2147/nss.S265946

Daghlas, I., Dashti, H.S., Lane, J., Aragam, K.G., Rutter, M.K., Saxena, R., et al. (2019). Sleep duration and myocardial infarction. *J Am Coll Cardiol.* 74, 1304-1314. doi: 10.1016/j.jacc.2019.07.022

Dashti, H.S., Redline, S.andSaxena, R. (2019). Polygenic risk score identifies associations between sleep duration and diseases determined from an electronic medical record biobank. *Sleep.* 42, doi: 10.1093/sleep/zsy247

Doherty, A., Smith-Byrne, K., Ferreira, T., Holmes, M.V., Holmes, C., Pulit, S.L., et al. (2018). Gwas identifies 14 loci for device-measured physical activity and sleep duration. *Nat Commun.* 9, 5257. doi: 10.1038/s41467-018-07743-4

Gao, X., Yang, X.C., Meng, L.X., Sun, H.L.andWang, T. (2020). [causal relationship between sleep and coronary artery disease: A mendelian randomization study]. *Zhonghua Liu Xing Bing Xue Za Zhi.* 41, 611-614. doi: 10.3760/cma.j.cn112338-20190624-00462

He, Q., Sun, H., Wu, X., Zhang, P., Dai, H., Ai, C., et al. (2017). Sleep duration and risk of stroke: A dose-response meta-analysis of prospective cohort studies. *Sleep Med.* 32, 66-74. doi: 10.1016/j.sleep.2016.12.012

Liao, L.Z., Li, W.D., Liu, Y., Li, J.P., Zhuang, X.D.andLiao, X.X. (2020). Causal assessment of sleep on coronary heart disease. *Sleep Med.* 67, 232-236. doi: 10.1016/j.sleep.2019.08.014

Liu, T.Z., Xu, C., Rota, M., Cai, H., Zhang, C., Shi, M.J., et al. (2017). Sleep duration and risk of all-cause mortality: A flexible, non-linear, meta-regression of 40 prospective cohort studies. *Sleep Med Rev.* 32, 28-36. doi: 10.1016/j.smrv.2016.02.005

Lu, H., Wu, P.F., Li, R.Z., Zhang, W.andHuang, G.X. (2020). Sleep duration and stroke: A mendelian randomization study. *Front Neurol.* 11, 976. doi: 10.3389/fneur.2020.00976

Pienaar, P.R., Kolbe-Alexander, T.L., Van Mechelen, W., Boot, C.R.L., Roden, L.C., Lambert, E.V., et al. (2021). Associations between self-reported sleep duration and mortality in employed individuals: Systematic review and meta-analysis. *Am J Health Promot.* 35, 853-865. doi: 10.1177/0890117121992288

Titova, O.E., Michaëlsson, K.andLarsson, S.C. (2020). Sleep duration and stroke: Prospective cohort study and mendelian randomization analysis. *Stroke.* 51, 3279-3285. doi: 10.1161/strokeaha.120.029902

Wang, D., Li, W., Cui, X., Meng, Y., Zhou, M., Xiao, L., et al. (2016). Sleep duration and risk of coronary heart disease: A systematic review and meta-analysis of prospective cohort studies. *Int J Cardiol.* 219, 231-9. doi: 10.1016/j.ijcard.2016.06.027

Zhao, J., Yang, F., Zhuo, C., Wang, Q., Qu, Z., Wang, Q., et al. (2021). Association of sleep duration with atrial fibrillation and heart failure: A mendelian randomization analysis. *Front Genet.* 12, 583658. doi: 10.3389/fgene.2021.583658

Zhuang, Z., Gao, M., Yang, R., Li, N., Liu, Z., Cao, W., et al. (2020). Association of physical activity, sedentary behaviours and sleep duration with cardiovascular diseases and lipid profiles: A mendelian randomization analysis. *Lipids Health Dis.* 19, 86. doi: 10.1186/s12944-020-01257-z
